# Collection of smartphone and internet addiction

**DOI:** 10.1186/s12888-023-04915-5

**Published:** 2023-06-14

**Authors:** Chung-Ying Lin, Zubair Ahmed Ratan, Amir H Pakpour

**Affiliations:** 1 Institute of Allied Health Sciences, College of Medicine, National Cheng Kung University, 1 University Rd, Tainan, 701401 Taiwan; 2 Department of Public Health, College of Medicine, National Cheng Kung University, Tainan, Taiwan; 3 Department of Occupational Therapy, College of Medicine, National Cheng Kung University, Tainan, Taiwan; 4 Biostatistics Consulting Center, National Cheng Kung University Hospital, National Cheng Kung University, Tainan, Taiwan; 5 School of Health and Society, University of Wollongong, Wollongong, NSW 2500 Australia; 6 Department of Nursing, School of Health and Welfare, Jönköping University, Jönköping, Sweden

## Abstract

The enigma of smartphone and internet addiction has plagued academics for the last decade, now scholars believe this behavior might have a substantial effect on human health and social issues. However, there are literature gaps. Thus, BMC Psychiatry works with us to launch the special collection “Smartphone and Internet Addiction”.

## Main text

Smartphone represents one of the most prominent technology advancements in the 21st century [1]. It extends the basic function of telephoning (i.e., for distant communication) to entertainment (e.g., gaming and video watching), social networking (e.g., social media use of *WhatsApp* and *Facebook*), financial management (e.g., online banking), learning and working (e.g., virtual meeting), etc. Although the powerful function of the technology is convenient for its users, a minority of smartphone users may have some problems of using smartphone properly [2, 3]. Because of the potential harms caused by the smartphone and internet, the scholarship focused on behavioral addictions has discussed this issue and pointed out some fundamental issue with smartphone use [4–6]. Accordingly, both American Psychiatric Association and World Health Organization have been aware of smartphone-related issues. Thus, they have attempted to define disorders relevant to the use of such technology. Their work has materialized in the *Diagnostic and Statistical Manual of Mental Disorders, Fifth Edition* (DSM-5) [7] and in the *International Classification of Diseases 11th Revision* (ICD-11) [8].

Some theories have been proposed to understand the development of smartphone and internet addiction [9–12]. For example, the *Interaction of Person-Affect-Cognition-Execution (I-PACE)* model suggests that individuals could use smartphones and/or internet as a coping strategy to overcome their difficulties in daily lives and to satisfy their emotional needs. However, when the individuals develop habitual and automatic behaviors of using smartphone/internet use due to the frequently used method of smartphone/internet, they are at risk of developing an addition to the mentioned. Moreover, the craving in smartphone/internet users may become stronger and cannot be resisted when the mentioned automatic loop is developed [9]. Some models propose that individuals with the tendency of narcissism can develop smartphone/internet addiction more easily than their counterparts : the *Self-Enhancement Model* describes that people use social media platforms to promote themselves.Those with high levels of narcissism are likely to use this method more frequently for self-promotion, which will, in turn, affect their dependency on smartphone and internet use [10]; the *Fit Model* indicates that people having high levels of narcissism perceive enjoyment from superficial social connects, which is easily achieved on the social media platforms, and thus they may frequently use and develop an addiction to them [12]; the *Trait Model* says that people with high levels of narcissism have extraversion characteristics to use more social media platform to make friends [11]. However, the mechanisms regarding smartphone/internet addiction development remain unclear because behavioral addiction is complex and additional evidence is needed to corroborate these proposed theories and models.

Apart from the mechanisms delineating the development of smartphone and internet addiction could be developed, healthcare providers and researchers are interested in how such behavioral addictions associate with health impairments. Indeed, current evidence from cross-sectional and longitudinal design has demonstrated the associations between smartphone/internet addiction and various health outcomes, such as psychological distress [13, 14], sleep problems [15, 16], and quality of life [17]. However, some scientific evidence reports positive effects of smartphone/internet addiction on health, such as the increase of physical activity [18, 19]. In this regard, the effects of smartphone and internet addiction on human being’s health needs additional evidence to provide a clear picture for healthcare providers in order to help them evaluate the benefits and harms of such addictions.

Given that smartphone technology continues improving and that the amount of people which possess them is steadily increasing, it is of utmost importance that we address the behavioral addictions related to smartphone use. For that reason, we are interested in collecting and cumulating scientific evidence associated with smartphone addiction and internet addiction across a variety of groups. In this regard, we expect that the present collection will be helpful in several aspects, such as clinical decision making, health programs design, and policy making, amongst others.

## Data Availability

Not applicable.
